# Validity and reliability of the Persian version of the Suboptimal Health Status Questionnaire among university staff in Iran

**DOI:** 10.7189/jogh.13.04162

**Published:** 2023-12-15

**Authors:** Erfan Ayubi, Salman Khazaei, Shiva Borzouei, Ali Reza Soltanian, Samereh Ghelichkhani, Fatemeh Karbin, Yuxiang Yan, Manshu Song, Cuihong Tian, Wei Zhang, Jing Sun, Wei Wang, Global Health Epidemiology Research Group (GHERG), Global Suboptimal Health Consortium (GSHC)

**Affiliations:** 1 Social Determinants of Health Research Center, Hamadan University of Medical Sciences, Hamadan, Iran; 2 Research Center for Health Sciences, Hamadan University of Medical Sciences, Hamadan, Iran; 3 Department of Endocrinology, School of Medicine, Hamadan University of Medical Sciences, Hamadan, Iran; 4 Modeling of Noncommunicable Diseases Research Center, Hamadan University of Medical Sciences, Hamadan, Iran; 5 Mother and Child Research Center, Hamadan University of Medical Sciences, Hamadan, Iran; 6 Student Research Committee, Hamadan University of Medical Sciences, Hamadan, Iran; 7 Beijing Key Laboratory of Clinical Epidemiology, School of Public Health, Capital Medical University, Beijing, China; 8 School of Medical and Health Sciences, Edith Cowan University, Joondalup, WA, Australia; 9 Clinical Research Center, First Affiliated Hospital of Shantou University Medical College, Shantou, Guangdong, China; 10 Centre for Cognitive Neurology, Department of Neurology, Beijing Tiantan Hospital, Capital Medical University, Beijing, China; 11 School of Medicine and Dentistry, and Institute for Integrated and Intelligent Systems, Griffith University, Gold Coast, Australia; 12 Center for Precision Health, Edith Cowan University, Joondalup, WA, Australia

## Abstract

**Background:**

Suboptimal Health Status Questionnaire-25 (SHSQ-25) is an established tool for measuring a precision health state between health and illness. The present study aims to assess the validity and reliability of a Persian version of SHSQ-25 (P-SHSQ-25) in a university staff Iranian population.

**Methods:**

A sample of 316 academic and supporting staff (163 males, age range from 23 to 64 years old) from Hamadan University of Medical Sciences, Hamadan, Iran was recruited in this population-based cross-sectional study with a questionnaire validation from Apri1 to October 2022. Forward-backward translation method was performed for the SHSQ-25 translation from English to Persian. Internal reliability, content, convergence, discriminative and construct validity of the P-SHSQ-25 were examined. The factorial structure of the P-SHSQ-25 across groups was examined using measurement invariant test.

**Results:**

In the translation process, the conceptual equivalence of the P-SHSQ-25 with the English version was confirmed. The item-content validity index and content validity ratio of all P-SHSQ-25 items were higher than the cut-off values of 0.70 and 0.62, respectively. Cronbach’s α was higher than 0.70 for all P-SHSQ-25 domains. The confirmatory factor analysis (CFA) showed the fitness of five factors on the data set (comparative fit index = 0.88, and root mean square error of approximation = 0.07). The CFA model fit did not change substantially across sex, age, occupation, economic status, and body mass index (Δ comparative fit index (CFI)<0.01).

**Conclusions:**

The P-SHSQ-25 can be used as a reliable and valid tool to measure health status for screening pre-chronic disease conditions in a primary care setting among Iranian population.

As early as in 1946, World Health Organization defined health as a state of complete physical, mental, and social well-being and not merely the absence of disease or infirmity [1]. However this definition has been criticised due to the consideration of “complete well-being” and the lack of operational values [2]. In most cases, subjective screening and monitoring questionnaires are applied to measure the different aspects of health status, such as, quality of life [3], mental health [4], diet and eating habits [5] and physical activity [6]. Although such questionnaires can improve clinical diagnosis, these tools are challenged due to the ambiguous nature of the questions, the time required to complete questionnaire, and the complex of interpreting the results. Therefore, it is necessary to develop more targeted and efficient tools to overcome these limitations.

To challenge the increase of the global health burden and in line with the efforts to develop appropriate questionnaires for measuring health precisely and to compensate for the limitations of some previous health measure tools, a tool, Suboptimal Health Status Questionnaire-25 (SHSQ-25), has been introduced recently [7]. SHSQ-25 has been created as a competitor and a leverage of existing instruments, owing to its simplicity, objectiveness of questions and easier scoring system [8,9]. Suboptimal Health Status (SHS) is a physical state between health and illness that is characterised by chronic fatigue along with a set of physical symptoms from the cardiovascular system, digestive system, immune system, and mental state, lasting for the past three months [10-12]. SHSQ-25 questionnaire is designed to identify people with health status which are not classified into a clinical phonotype defined by the International Classification of Disease [7]. SHS indicates a pre-chronic disease condition that can be associated with body weakness, decreased appetite, and lack of vitality [8]. It has become a major public health concern worldwide in the context of global health [11]. Suboptimal Health Status has been associated with various chronic diseases such as type 2 diabetes mellitus [13], cardiovascular disease (CVD), metabolic syndrome [14] and preeclampsia [15]. The correlation between ideal cardiovascular health metrics and CVDs can also be elucidated through the concept of SHS [16], e.g. previous study has reported a relationship between SHS and endothelial dysfunction [17]. In addition, several studies have reported evidence of relationship between SHS and objective biomarkers such as plasma cortisol [9], mRNA expression of glucocorticoid receptor α/β [9], plasma metabolites [18], N-glycosylation profiles [15], oxidative stress [18], telomere length [19] and intestinal microbiota [20].

The reliability and validity of SHSQ-25 in Chinese, English, Korean and Russian have been evaluated in three major ethnic groups: African, Asians and Caucasians [17,21,22]. For the present study, it is hypothesised that the Persian version of SHSQ-25 (P-SHSQ-25) is a valid and reliable tool to early identify people with non-communicable diseases (NCDs) in Iran. Therefore, this current study aims to assess the validity and reliability of a P-SHSQ-25 in an Iranian university staff population by focusing on the Persian language and lifestyle reflected by Iranian’s unique education, economic and occupation.

## METHODS

### Design and study population

This is a population-based cross-sectional study with a questionnaire validation at the Hamadan University of Medical Sciences, Hamadan, Iran. The study population included academic and supporting staff of various faculties, including vice-chancellors and staff from the teaching hospitals using a proportional quota sampling at the workplaces.

After explaining the study objectives and obtaining the informed consent, the participants were recruited in the study from April to October 2022. People with a history of chronic diseases, such as hypertension, diabetes, respiratory disease, CVDs, immunosuppression, hematological systems, and mental diseases, as well as those who use medications for chronic disorders were excluded.

### Sample size calculation

Rule of thumb was used to calculate the sample size of the study. A subjects-to-variables ratio of 10:1 or 15:1 is suggested for factor analysis to meet adequate statistical power [23]. There are 25 items in the SHSQ-25 of this current study. There should be at least 10 subjects for each item in the instrument being used. In total, at least 250 participants should be considered.

### Description of the SHSQ-25

The SHSQ-25 contains 25 questions which measures health status in five domains: fatigue (9 items), cardiovascular system (3 items), digestive system (3 items), immune system (3 items), and mental status (7 items). The questions are related to the state of health in the last three months. Using a 5-point Likert scale for the questions, the participants specify their health status as 1 (never or almost never), 2 (occasionally), 3 (often), 4 (very often), and 5 (always). The total score of SHSQ-25 ranges from 0 to 100 points after the raw scores of 1 to 5 on the questionnaire are recoded as 0 to 4. The higher scores indicate poorer health status [7].

### Translation of SHSQ-25 from English into Persian

The forward-backward translation was applied to creation of the P-SHSQ-25. In the forward translation process, the original version of the SHSQ-25 tool was translated into Persian language by two independent authors (EA and SK). Any disagreement in the translations of technical terms and medical jargon was resolved by the third author (SB). In the backward translation process, the Persian version was translated into English by a professional translator who was a faculty member of the university and was blinded to the original English version of SHSQ-25. The translated English version was compared with the original English version of SHSQ-25. By the study authors, the P-SHSQ-25 was proofed and finalised.

### Content validity

The content validity of the P-SHSQ-25 was evaluated using the content validity index (CVI) and content validity ratio (CVR) [24]. For this reason, ten members of the university faculty consisting of internal medicine specialists, community medicine specialists, and epidemiologists as the expert panel was asked to rate the relevance, clarity, and simplicity of the items of P-SHSQ-25 on a Likert scale, e.g. 1 (not relevant), 2 (somewhat relevant), 3 (quite relevant), 4 (highly relevant). Item-CVI was calculated using dividing the number of experts who rated the item as 3 and 4 by the total number of the expert panel. The value of 0.70 to 0.80 of item-CVR is acceptable, but needs for revision, while the value of 0.80 and above is appropriate without further revision [25]. Moreover, the expert panel was asked to score the essential of the items on a Likert scale from 1 to 3 as 1) not necessary, 2) useful but not essential, and 3) essential, respectively. Content validity ratio was calculated according to the following equation:



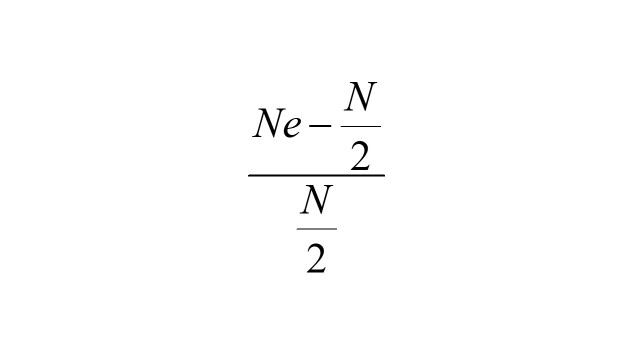



where, Ne is the number of experts who have selected the “essential” choice and N is the total number of the expert panel.

The optimal cut-off CVR was determined accrediting to the Lawshe Table. Since the number of panelists in this current study was 10, the minimum acceptable cut-off for CVR was estimated to be 0.62 [24].

### Internal consistency

Cronbach's α coefficient was used to evaluate the internal consistency of the P-SHSQ-25 as a whole and for its five domains. Cronbach’s α was estimated to assess the effect of each item on internal consistency of P-SHSQ-25 domains. Moreover, the internal consistency of P-SHSQ-25 was checked using the split-half reliability coefficient. The optimal range of Cronbach's α and split-half coefficients are the value of 0.70 or above.

### Discriminative validity

The significant difference in the score of the P-SHSQ-25 and its domains in different groups indicate the measure of discriminative validity. The Shapiro-Wilk test was used to evaluate the normal distribution. The data following skewed distribution were presented by a median with interquartile range (P25, P75). The Kruskal-Wallis test or Mann-Whitney U test were used to compare the median scores of questionnaires according to gender, age group, type of occupation, socioeconomic status, and body mass index (BMI). Asset-based wealth index was used to measure economic status. Participants were asked to identify household assets and the state of the house including car, washing machine, side-by-side refrigerator, dishwashing machine, microwave, play station/Xbox console, >49-inch smart TV, type of home ownership (owner or tenant) and the number of the room. Asset-based wealth index was constructed using principal component analysis. The first component with a higher explained variance was extracted and the predicted score from the first component was divided into quintiles.

### Convergent validity

To evaluate the convergence validity of P-SHSQ-25, the correlational value of P-SHSQ-25 and its five domains was assessed with another established questionnaire with similar health focus but with different emphasis domains. The World Health Survey (WHS) individual questionnaire measures a person's health status in eight domains: mobility, self-care, cognition and perception, interpersonal relationships, vision status, sleep status, pain and mental status using ten questions with Likert scale [26]. The correlation between P-SHSQ-25 and its domain with WHS individual questionnaire was evaluated using Pearson correlation.

### Construct validity

Exploratory factor analysis (EFA) via varimax rotation was applied to assess the construct of P-SHSQ-25. Eigenvalues greater than 1.00 were the criterion for extraction of the factor. P-SHSQ-25 items with factor loading 0.40 were retained in the extracted factor. The construct validity of the P-SHSQ-25 was examined from the confirmatory factor analysis (CFA) through the maximum likelihood estimation method. Regarding factor loadings, the average variance extracted (AVE) was estimated. The average variance extracted represents the amount of variance of a variable that is explained by latent unobserved variable compared to the amount of variance due to measurement error [27]. The amount of shared variance among the items of a given domain was evaluated using composite reliability (CR). The AVE and CR range from 0.00 to 1.00 and AVE of greater than 0.50 and CR of greater than 0.70 represent optimal cut-off [28]. The goodness-of-fit indexes were as follows: comparative fit index (CFI), goodness-of-fit index (GFI), normed fit index (NFI), Tucker-Lewis index (TLI) with the minimum acceptable cut-off of greater than 0.90 and root mean square error of approximation (RMSEA) and root mean square residual (RMR) and Standardized RMR (SRMR) with values lower than 0.05 indicate better fitness. Measurement invariant (MI) was used to evaluate the stability of the 5-factor structure of the SHSQ-25 across different groups. To test for MI, three constrained CFA models were compared with a model without any equality constraints. Four CFA models were as follows: A) configural invariance model without any equality constraints, B) metric invariance model with assuming equality of factor loading across groups but allowing diversity in intercepts between groups (weak invariance), C) scalar invariance model with assuming equality of both the factor loadings and intercepts across groups (strong invariance) and D) strict invariance model with assuming equality the factor loadings, intercepts and residual variances across groups (strict invariance). Weighted least squares means and variance adjusted estimation was used to estimate parameters. Non-significant results for χ^2^ difference (Δ χ^2^) test indicate the model fitness did not change after constraining. In accordance with previous recommendation, ΔCFI<0.01 indicates an evidence in favor of holding invariance assumption [29]. All statistical tests were considered significant if *P*-value less than 0.05. All statistical analyses were conducted using SPSS 22.0, Amos 24.0, and R 4.1.3.

## RESULTS

A total of 380 questionnaires were distributed during seven months (Apri1-October 2022) and 316 completed questionnaires were received (response rate = 83%) and included in the final analyses. Overall, 163 (51.58%) questionnaire respondents were male, and range of age was 23 to 64 years. The median (P25, P75) of total P-SHSQ-25 scores was 21 (15-31) and scores of fatigue, cardiovascular system, immune system, digestive system and mental status domains were 9 (6-13), 1 (0-3), 3 (2-4), 2 (0-3) and 6 (4-9), respectively.

The CVI values for relevance, clarity, and simplicity of all P-SHSQ-25 items were higher than 0.70. In a panel of the study authors, the SHSQ-25 items with item-CVI between 0.70 and 0.80 were edited again. The CVRs of all items of P-SHSQ-25 were higher than the acceptable cut-off of 0.62. The data are presented in Table S1 in the **Online Supplementary Document**.

The median of the total scores of the P-SHSQ-25 was significantly higher in female than in male (*P* < 0.01). Likewise, the highest score of SHS and its domains were observed in the age group of 35-45 years which comprised of 47.19% of the study population. The participants with higher economic status statistically and significantly were less likely to have higher score of total P-SHSQ-25 and two domains of immune system and mental status than those with lower economic status. As compared to academic staff, non-academic staff had a significant higher median score for digestive tract domain. The score of SHSQ-25 and its domains did not statistically vary according to BMI. The total scores of the P-SHSQ-25 and its domains according to the background characteristics of the questionnaire respondents are showed in detail in the Table S2 in the **Online Supplementary Document**.

**Figure 1:**
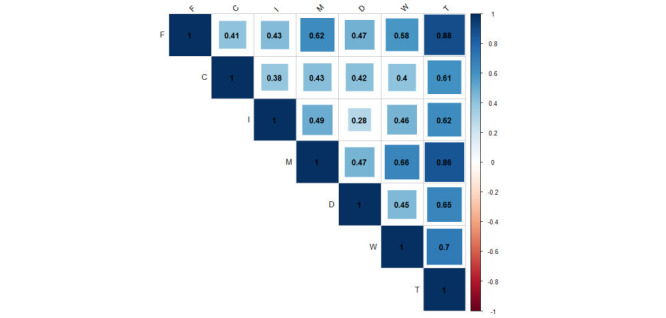
Correlation between Persian version of SHSQ-25 (P-SHSQ-25) and its domains and World Health Survey (WHS) individual questionnaire. F – fatigue, M – mental status, C – cardiovascular system, D – digestive system, I – immune system, T – total score of P-SHSQ-25, W – World Health Survey (WHS) individual questionnaire

**Table 1** shows the results of the reliability analysis. The Cronbach’s α value for fatigue, cardiovascular system, digestive tract, immune system, and mental status domains were 0.89, 0.74, 0.82, 0.78, and 0.87, respectively. The observed values of higher than 0.70 indicated internal consistency of all items of P-SHSQ-25. The split-half reliability coefficient was 0.83. **Figure 1** shows the Pearson correlation coefficient among P-SHSQ-25 and its domains with WHS individual questionnaire. There was significant correlation between WHS individual questionnaire and the fatigue (Pearson correlation coefficient = 0.58), cardiovascular system (0.40), Immune system (0.46), Digestive tract (0.45) and mental status (0.66) and total SHSQ-25 (0.70). All the presented correlations less than 0.001were considered statistically significant.

**Table 1:** The results of internal reliability analysis of the Persian version of SHSQ-25 (P-SHSQ-25)*

Domain (Cronbach’s α)	Item number	Corrected item-total correlation	Cronbach’s α if item is deleted
Fatigue (0.89)	SHSQ-1	0.67	0.87
	SHSQ-2	0.73	0.87
	SHSQ-3	0.71	0.87
	SHSQ-4	0.64	0.88
	SHSQ-5	0.45	0.89
	SHSQ-6	0.62	0.88
	SHSQ-8	0.69	0.87
	SHSQ-9	0.66	0.87
	SHSQ-10	0.62	0.88
Cardiovascular system (0.74)	SHSQ-11	0.58	0.65
	SHSQ-12	0.52	0.71
	SHSQ-13	0.60	0.61
Digestive tract (0.82)	SHSQ-14	0.67	0.77
	SHSQ-15	0.66	0.78
	SHSQ-16	0.72	0.73
Immune system (0.78)	SHSQ-7	0.65	0.67
	SHSQ-17	0.53	0.82
	SHSQ-25	0.69	0.62
Mental status (0.87)	SHSQ-18	0.62	0.88
	SHSQ-19	0.62	0.88
	SHSQ-20	0.71	0.87
	SHSQ-21	0.73	0.87
	SHSQ-22	0.77	0.86
	SHSQ-23	0.74	0.87
	SHSQ-24	0.67	0.88

*Persian version of SHSQ-25 (P-SHSQ-total) = 0.93; split-half reliability coefficient = 0.83.

**Figure 2:**
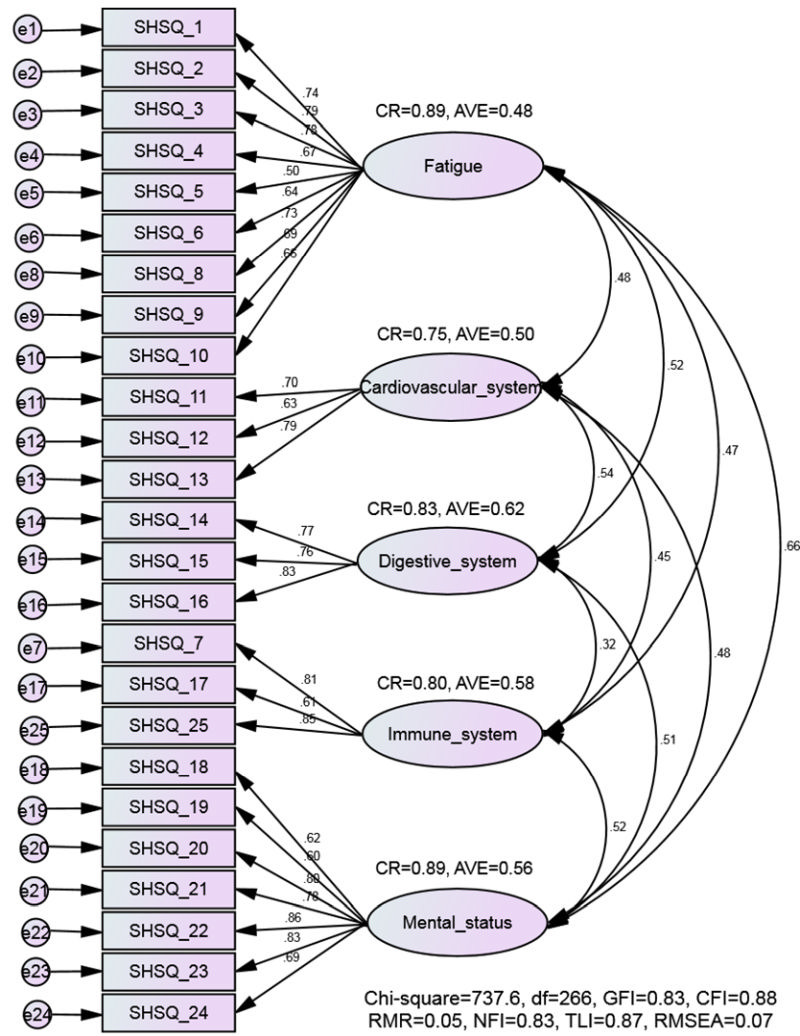
Confirmatory factor analysis of the Persian version of SHSQ-25 (P-SHSQ-25). Correlation between the item and the P-SHSQ-25 domains (factors) is strong (factor loading >0.50). All of goodness of fit indexes (e.g. CFI  0.90 and RMSEA  0.05) indicate the fitness of the hypothesised model on the data. The Convergent validity indexes are above the minimal threshold (CR>0.70 and AVE>0.50). CR – composite reliability, AVE – average variance extracted, df – degree of freedom, RMR – root mean square residual, GFI – goodness-of-fit index, NFI – normed fit index, TLI – Tucker-Lewis index, CFI – Comparative fit index, RMSEA – root mean square error of approximation

**Table 2** shows the results of the EFA. The 25 items of P-SHSQ-25 were reduced to 5 factors with an eigenvalue of >1.00 which explained 63.62% of the variance of the data set. The pattern of loading of the items on factors was similar to the original SHSQ-25 version but only the item SHSQ5 about dizziness was not loaded on the five domains of P-SHSQ-25. **Figure 2** presents the confirmatory factor model of P-SHSQ-25 and its five domains. All P-SHSQ-25 items had factor loading higher than 0.40 on the corresponding domain. The CR values for the five domains of P-SHSQ-25 were higher than 0.70 indicating good construct reliability. The AVE values for fatigue, cardiovascular system, digestive tract, immune system, and mental status were 0.48, 0.50, 0.62, 0.58, and 0.56, respectively indicating evidence for moderate-good construct validity. The model fit summary was as follows: RMR = 0.05, SRMR = 0.06, GFI = 0.83, NFI = 0.83, TLI = 0.87, CFI = 0.88 and RMSEA = 0.07. The results of MI tests and model comparisons showed not statistically significant for Δ χ^2^ (*P* > 0.05) and Δ CFI were also lower than the cut-off value of 0.01. This indicates factor loadings, intercepts and residuals can be assumed to be equal across the groups (**Table 3**). The P-SHSQ-25 is provided in Appendix A in the **Online Supplementary Document**.

**Table 2:** Results of exploratory factor analysis of the Persian version of SHSQ-25 (P-SHSQ-25)

Item number	Factor 1	Factor 2	Factor 3	Factor 4	Factor 5
SHSQ-1	0.67*	0.36	0.05	0.15	0.02
SHSQ-2	0.70*	0.25	0.17	0.18	0.12
SHSQ-3	0.70*	0.38	0.09	0.08	0.07
SHSQ-4	0.71*	0.11	0.24	0.10	<0.01
SHSQ-5	0.33	0.29	0.37	-0.03	0.23
SHSQ-6	0.68*	0.24	0.05	-0.05	0.08
SHSQ-7	0.11	0.26	0.09	0.81*	0.04
SHSQ-8	0.72*	0.09	0.12	0.25	0.19
SHSQ-9	0.72*	0.24	0.06	0.09	0.09
SHSQ-10	0.66*	0.02	0.26	0.15	0.24
SHSQ-11	0.13	0.08	0.05	0.23	0.77*
SHSQ-12	0.11	0.17	0.15	0.04	0.76*
SHSQ-13	0.14	0.17	0.25	0.12	0.73*
SHSQ-14	0.18	0.20	0.77*	0.03	0.14
SHSQ-15	0.20	0.17	0.80*	0.06	0.10
SHSQ-16	0.13	0.16	0.82*	0.16	0.17
SHSQ-17	0.21	0.18	0.07	0.66*	0.21
SHSQ-18	0.26	0.52*	0.16	0.26	0.19
SHSQ-19	0.23	0.52*	0.27	0.12	0.28
SHSQ-20	0.22	0.74*	0.16	0.18	0.08
SHSQ-21	0.20	0.80*	0.07	0.11	0.05
SHSQ-22	0.21	0.80*	0.17	0.12	0.12
SHSQ-23	0.14	0.80*	0.13	0.12	0.17
SHSQ-24	0.33	0.65*	0.12	0.18	0.03
SHSQ-25	0.15	0.18	0.06	0.84*	0.14
Eigenvalue	9.41	1.92	1.83	1.59	1.14
% explained variance	37.64	7.68	7.33	6.38	4.57

*The factor loading greater than 0.40.

**Table 3:** Measurement Invariance Analysis of Five Factor Structure of the P-SHSQ-25 across Subgroups of the Questionnaire Respondents

Characteristics	Chi-square	*df*	Δ Chi-square	Δ *df*	*P*-value	CFI
**Sex**						
A*	238.86	484				0.99
B†	320.63	503	24.89	19	0.16	0.98
C‡	332.51	522	23.11	19	0.23	0.98
D§	360.66	546	46.92	24	<0.01	0.98
**Age**						
A*	327.39	726				0.98
B†	461.86	764	43.99	38	0.23	0.98
C‡	481.92	802	41.94	38	0.30	0.98
D§	517.31	850	61.90	48	0.08	0.98
**Occupation**						
A*	210.95	484				
B†	266.80	503	18.63	19	0.48	1.00
C‡	280.64	522	29.76	19	0.05	1.00
D§	295.80	546	27.61	24	0.27	1.00
**Economic status**						
A*	537.22	1210				0.98
B†	818.36	1286	81.63	76	0.31	0.98
C‡	852.37	1362	71.17	76	0.63	0.98
D§	946.33	1458	140.06	96	<0.01	0.97
**BMI‖**						
A*	208.86	484				1.00
B†	269.12	503	20.45	19	0.36	1.00
C‡	279.19	522	20.90	19	0.34	1.00
D§	295.36	546	30.28	24	0.17	1.00

*df* – degree of freedom, CFI – comparative fit index, BMI – body mass index

*Configural invariance model.

†Metric invariance model.

‡Scalar invariance model.

§Strict invariance model.

‖Due to limited sample size, only normal and overweight subjects were included in the analysis.

## DISCUSSION

To the best of our knowledge, this is the first study evaluating the validity and reliability of the P-SHSQ-25 in an Iranian population. Our results indicated that the P-SHSQ-25 has a good validity and an internal reliability among the Iranian adult population.

Although the concept of SHSQ-25 is based on traditional Chinese medicine, studies showed the reliability and validity of English and Chinese Versions of SHSQ-25 [30,31], and also in other languages such as Russian [17] and Korean [22]. Consistent with the above-mentioned studies, our current study implied the reliability and validity of the P-SHSQ-25 in Iranian population.

In this current study, the properties of the SHSQ-25 were examined in an Iranian population who ethnically differs from previously studied African, Asian and Caucasian population [17,21,22]. Evaluating the SHSQ-25 in the different population may provide evidence of cross-cultural validity of SHSQ-25 from the global health perspective [11]. Our study is consistent with previous studies [21,22] providing good evidence in favor of reliability (e.g. Cronbach's α >0.70) and interrelationship among SHSQ-25 domains (e.g. Pearson correlation coefficient >0.70). However, there were differences in term goodness of fit indicators in our study when compared with previous studies. For example, in our study the goodness of fit indicators including RMSEA, CFI, GFI and TLI were 0.07, 0.88, 0.83 and 0.87, respectively and also CR measures for the all SHSQ-25 domains were above 0.70. In a study by Adua et al. [21] among African population, CR measures for the all SHSQ-25 domains were below 0.70 except for fatigue and mental health. Moreover, in the aforementioned study the goodness of fit indicators including RMSEA, CFI, GFI and TLI were reported 0.10, 0.81, 0.78 and 0.79, respectively. In another study in Korean population [22], the RMSEA, GFI and adjusted GFI were 0.07, 0.93 and 0.91, respectively. Here, one could think that the fitness of the SHSQ-25 constructs in Iranian population is more optimal than that in African population and less than in East Asian populations.

In contradict to the previous studies [21,22], we further confirmed the content and convergent validity of the SHSQ-25 after a cross-cultural translation process. In our current study, the factor loadings were modest and statistically significant, providing evidence for convergence validity among people who with diverse socioeconomic background in response to SHS scoring allocated in the SHSQ-25 constructs [32]. In addition, we examined the correlation of P-SHSQ-25 with another established tool, WHS, to check the complementarity between the P-SHSQ-25 and WHS, and therefore, evidenced the convergent validity of the P-SHSQ-25. Since validity of the P-SHSQ-25 has been evaluated in different ways, it can be applied to facilitate implementation of global health concept in Persian as a mother tongue version to overcome the language obstacle of English in Iranian population, exactly like that respective native language versions of SHSQ-25 have been effectively used as a health measure tool in China, Korea, Russia and English speaking countries [11,17,21,22].

Following the improvement in life expectancy and modernisation of Iranian’s lifestyle, NCDs become a pivotal challenge for the Iranian health care system [33]. Health statistics indicate NCDs are responsible of 81% of deaths in Iran [34,35]. Deaths from NCDs can be alleviated by providing the optimal health intervention to the right patient with right dosage and right therapy at the right time [36]. SHSQ-25 is such a tool can be used under this precision health strategy and plays significant role in NCDs’ prevention and control from the context of global health [37]. Primary Medicare essentially requires actions to identify individuals with SHS.

The descriptive measures of the P-SHSQ-25 in this current study on university academic and general supporting staff were in line with the result from a SHS study among nurses [38], but were lower than that from the SHS studies in other general populations [7,21], indicating occupation and social status are important contributors for SHS assessment.

Cronbach's α coefficients of the items SHSQ-11 (breathlessness) and SHSQ-13 (heart palpitations) loaded on the cardiovascular system domain and the items SHSQ-25 (catch colds) and SHSQ-7 (sore throat) loaded on the immune system were between 0.60 and 0.70 respectively, indicating modest internal reliability and chance of measurement error. Response bias styles such as acquiescence/disacquiescence, mid/mild point response, or extreme response for Likert-type rating scale are the main source of measurement error [39]. For example, in this current study there was disacquiescence style for the item SHSQ-11 (63.6% selected choice of 0 “never or almost never”) and a mid-point response style for SHSQ-25 (63% selected choice of 1 “occasionally”). The response rate bias can influence other measures such as factor loading, e.g. the item SHSQ5 “dizziness” was not loaded on the fatigue domain. The factors loading of dizziness in the studies in Chinese [7], Ghanaians [21] and Korean [22] were 0.39, 0.62 and 0.68, respectively.

From the perspectives of global health, SHS can provide a window of opportunity to identify individual predisposition before the onset of a NCD, and thus provide targeted prevention measures and create personalised/precisive health intervention [11]. To collect baseline information of SHS among Iranian adult population, a practical questionnaire in a local language such as the P-SHSQ-25, can be of vital help.

Although more participants than we need (oversample, 15 participants per item) have been recruited and several strategies such as incentives have been employed to reduce the dropout rate and enhance the overall quality of the findings in this study, we encountered a dropout rate of 17%. Several factors might have contributed to this dropout rate: (1) some participants didn’t complete the questionnaire owing to disengagement, loss of interest, or time constraints; (2) we have withdrawn some participants from the study because they failed to meet inclusion criteria (e.g. having chronic diseases) after the study began. However, a dropout rate, missing data, response bias, or attrition rate of less than 20% is relatively common and generally considered acceptable in observational studies, while this value >20% poses threats to validity [40].

The Persian version of SHSQ-25 is necessary but still has its limitation and not sufficient. A further effort to support the subjective health measure with objective health measure by integrating the Persian version of SHSQ-25 with anthropometric and biochemistry objective biomarkers, such as blood pressure, BMI, plasma glucose, plasms lipids and glycans is warranted from the predictive, preventive and personalised medicine standpoints [41-43].

In addition, the results of this current study can be influenced by self-reporting, e.g. it was expected the scores of P-SHSQ-25 and its domain were significantly varied according to BMI category, but non statistically significant was found in the current study. Second, although the goodness-of-fit indexes was close to optimal cut-off, a degree of measurement errors precluded that these indexes reach to optimal cut-off. Third, as the P-SHSQ-25 is a screening tool of heath condition for chronic health conditions, its validity for the prediction of future outcomes (predictive validity) can be evaluated more effectively in a longitudinal cohort study to overcome the limitations that causal relationship was not able to be inferred in this current study and the test-retest reliability of the P-SHSQ-25 could not be performed.

## CONCLUSIONS

We have created a Persian version of SHSQ-25 (P-SHSQ-25) and constructed its validity in university staff Iranian population. The P-SHSQ-25 can be used to measure health status before the onset of chronic disease in a primary care setting among the Iranian adult population. The combination of the P-SHSQ-25 and objective health measures is encouraged to be integrated to measure health status precisely. Further prospective large population-based studies are required to confirm the predictive validity and reliability of this novel generic tool for its application in general Iranian population.

## Additional material


Online Supplementary Document
