# Supplementary material for: Validity and reliability of the Persian version of the Suboptimal Health Status Questionnaire among university staff in Iran
Source: J Glob Health. 2023 Dec 15;13:04162. doi: 10.7189/jogh.13.04162 (PMC10722246; doi:10.7189/jogh.13.04162)
Supplement: Online Supplementary Document [file jogh-13-04162-s001.pdf]

Online Supplementary Document

Table S1. The results of Content Validity Analysis of the P-SHSQ-25

Item no.	Questions	CVI (%)			CVR (%)
		Relevance	Clarity	Simplicity	Essential
SHSQ-1	Were exhausted without greatly increasing your physical activity?	100.0	90.9	90.9	100.0
SHSQ-2	Experienced fatigue that could not be substantially alleviated by rest?	100.0	100.0	100.0	100.0
SHSQ-3	Were lethargic when working?	81.8	90.9	100.0	81.8
SHSQ-4	Suffered from headaches?	90.9	90.9	100.0	63.6
SHSQ-5	Suffered from dizziness?	100.0	100.0	100.0	81.8
SHSQ-6	Eyes ached or were tired?	90.9	100.0	100.0	63.6
SHSQ-7	Suffered from a sore throat?	72.7	90.9	90.9	63.6
SHSQ-8	Muscles or joints felt stiff?	63.6	90.9	90.9	81.8
SHSQ-9	Have pain in your shoulder/neck/waist?	90.9	100.0	100.0	81.8
SHSQ-10	Have a heavy feeling in your legs when walking?	72.7	90.9	90.9	63.6
SHSQ-11	Felt out of breath while sitting still?	90.9	90.9	90.9	81.8
SHSQ-12	Suffered from chest congestion?	81.8	72.7	81.8	63.6
SHSQ-13	Were bothered by heart palpitations?	90.9	100.0	100.0	81.8
SHSQ-14	Appetite was poor?	90.9	100.0	100.0	63.6
SHSQ-15	Suffered from heartburn?	90.9	100.0	100.0	81.8
SHSQ-16	Suffered from nausea?	72.7	90.9	90.9	63.6
SHSQ-17	Could not tolerate cold environments?	81.8	90.9	90.9	63.6
SHSQ-18	Had difficulty falling asleep?	100.0	100.0	100.0	100.0
SHSQ-19	Had trouble with waking up during night?	90.9	100.0	100.0	81.8
SHSQ-20	Had trouble with your short-term memory?	100.0	90.9	81.8	81.8
SHSQ-21	Could not respond quickly?	72.7	72.7	72.7	81.8
SHSQ-22	Had difficulty concentrating?	90.9	81.8	72.7	81.8
SHSQ-23	Were distracted for no reason?	90.9	100.0	100.0	81.8
SHSQ-24	Felt nervous or jittery?	100.0	90.9	81.8	100.0
SHSQ-25	Caught a cold in the past 3 months?	81.8	100.0	100.0	81.8

SHSQ, suboptimal health status questionnaire; CVI, content validity index; CVR, content validity ratio.

Table S2. Characteristics of Questionnaire Respondents according to the Domain and Total Scores of the P-SHSQ-25

Variables	N (%)	Fatigue	Cardiovascular system	Immune system	Digestive tract	Mental status	Total SHS
**Sex**							
Male	163 (51.58)	8 (5-12)	1 (0-3)	3 (2-3)	1 (0-3)	6 (4-8)	18 (13-27)
Female	153 (48.42)	10 (8-15)	1 (0-3)	3 (2-4)	2 (1-3)	7 (5-10)	25 (18-34)
p-value		<0.01	0.43	0.03	0.08	<0.01	<0.01
**Age** (years)							
<35	98 (32.34)	9 (5-13)	1 (0-2)	3 (2-4)	2 (0-3)	6 (3-9)	20 (13-30)
35-45	143 (47.19)	10 (6-14)	2 (0-3)	3 (2-4)	2 (1-3)	7 (5-10)	23 (17-34)
>45	62 (20.46)	8 (5-10)	1 (0-2)	2.50 (1-3)	1 (0-3)	6 (4-8)	19 (13-24)
p-value		0.02	0.11	0.02	0.04	<0.01	<0.01
**Occupation**							
Academic staff	83 (26.27)	9 (6-14)	1 (0-3)	3 (2-4)	1 (0-3)	7 (5-9)	21 (14-30)
Non-academic staff	233 (73.73)	9 (5-13)	1 (0-3)	3 (2-4)	2 (1-3)	6 (4-9)	21 (15-31)
p-value		0.78	0.52	0.85	<0.01	0.64	0.77
**Economic status**							
Q1	54 (20.30)	9 (6-14)	1 (0-2)	3 (2-3)	2 (1-3)	5 (3-8)	19 (14-31)
Q2	53 (19.92)	10 (8-14)	1 (0-3)	3 (3-4)	2 (1-3)	7 (5-10)	25 (18-36)
Q3	53 (19.92)	10 (5-14)	2 (0-3)	3 (2-4)	2 (1-3)	6 (5-10)	23 (15-35)
Q4	53 (19.92)	8 (4-11)	1 (0-2)	3 (2-4)	1 (0-3)	6 (4-8)	19 (14-25)
Q5	53 (19.92)	9 (6-12)	1 (0-2)	2 (1-3)	1 (0-3)	6 (4-8)	19 (14-29)
p-value		0.06	0.73	0.05	0.43	0.05	0.05
**BMI** (kg/m^2)							
Underweight	13 (4.15)	11 (8-14)	1 (0-2)	3 (1-3)	2 (1-3)	6 (1-13)	24 (16-38)
Normal weight	142 (49.52)	9 (5-13)	1 (0-3)	3 (2-3)	2 (0-3)	6 (4-8)	20 (14-28)
Overweight	139 (43.45)	9 (6-14)	2 (0-3)	3 (2-4)	2 (0-3)	7 (4-10)	22 (16-34)
Obese	22 (7.03)	9.50 (4-13)	1 (0-3)	3 (2-4)	2 (1-3)	6.50 (4-11)	21 (14-36)
p-value		0.32	0.15	0.09	0.75	0.17	0.23

Q, quintile; BMI, body mass index. Data are shown as number (percentage) or median (P25, P75).

Appendix A. Persian version of SHSQ-25

ردیف	لطفا به سوالات زیر با توجه به 3 ماه گذشته پاسخ دهید	هرگز یا تقریبا هرگز 1	گهگاهی 2	اغلب 3	بیشتر اوقات 4	همیشه 5
1	هر چند وقت بدون افزایش در فعالیت فیزیکی، احساس خستگی کرده اید؟					
2	هر چند وقت دچار خستگی شده اید که با استراحت کردن برطرف نشده است؟					
3	هر چند وقت در زندگی روزمره خود دچار حالت سستی و بیحالی شده اید؟					
4	هرچندوقت از سردرد رنج برده اید؟					
5	هر چند وقت از سرگیجه رنج برده اید؟					
6	هرچند وقت دچار احساس درد و خستگی در چشم ها شده اید؟					
7	هر چند وقت از گلودرد رنج برده اید؟					
8	هر چند وقت در ماهیچه ها یا مفاصل خود احساس سفتی و گرفتگی کرده اید؟					
9	هرچندوقت در پشت، شانه و گردن خود درد داشته اید؟					
10	هرچندوقت احساس سنگینی و تورم در پاها هنگام راه رفتن داشته اید؟					
11	هرچند وقت احساس تنگی نفس در هنگام استراحت داشته اید؟					
12	هر چند وقت از احتقان قفسه سینه رنج برده اید؟					
13	هر چند وقت تپش قلب شما را آزار داده است؟					
14	هر چند وقت دچار کم اشتهایی شده اید؟					
15	هر چند وقت از سوزش معده و سردل رنج برده اید؟					
16	هر چند وقت از تهوع رنج برده اید؟					
17	هرچند وقت تحمل سرما برای شما دشوار بوده است؟					
18	هر چند وقت مشکل در به خواب رفتن داشته اید؟					
19	هر چند وقت با مشکل از خواب پریدن در طول شب رو به رو شده اید؟					
20	هر چند وقت حافظه کوتاه مدت شما دچار مشکل شده است؟					

21 هر چند وقت هنگام پاسخ سریع به موقعیت ها و تصمیم گیری دچار مشکل شده اید؟

22 هر چند وقت دچار مشکل در تمرکز شده اید؟

23 هر چند وقت بی دلیل دچار حواس پرتی شده اید؟

24 هرچند وقت حالت دلهره و عصبی شدن داشته اید؟

25 هر چند وقت دچار سرماخوردگی شده اید؟
